# THE AGE-FRIENDLY COMMUNITY ENVIRONMENT FACTORS CONTRIBUTING TO LIFE SATISFACTION OF OLDER ADULTS LIVING ALONE

**DOI:** 10.1093/geroni/igac059.2303

**Published:** 2022-12-20

**Authors:** Mi Sun Choi, Hyunmin Lee

**Affiliations:** Silla University, Busan, Pusan-jikhalsi, Republic of Korea; Seoul, Seoul, Seoul-t'ukpyolsi, Republic of Korea

## Abstract

Age-friendliness studies in Korea have investigated a few subsets of the local community environments for older adults’ well-being. Little is known about comprehensively understanding which aspects in community contexts can help older individuals living alone improve their life satisfaction. To address the knowledge gaps, this study utilized the raw data from the 2020 National Survey of Older Koreans, and responses of 3,112 older adult single-person households were investigated. From an ecological perspective, hierarchical regression analysis was performed by distinguishing the community environments into three categories of the World Health Organization’s age-friendly environments: physical, social, and service environments. The results showed that the perception of the physical environment (housing, living facilities, and space) was positively associated with life satisfaction. Second, the social environment (social involvement, neighbor interaction, respect for older individuals, and political activity) was positively related to life satisfaction. Third, perceived service environment (difficulty in utilizing service) was negatively related to life satisfaction. Based on the findings, we proposed political and practical recommendations. Specifically, as a physical environment aspect, effective budget allocation and policy attention should be required for the autonomy and independence of older adults living alone in their daily lives by ensuring that housing and circumstances are suitable for aging-in-place. As a societal component, initiatives must be established to promote participation in decision-making processes that result in a more favorable social perception through social inclusion. Finally, for the service environment, we must advocate for increased accessibility to community supportive services (mobility, health care, food delivery, communication, and information).

